# Wnt Signaling-Related Biomarkers in Gestational Diabetes Mellitus: Diagnostic Performance and Integrated Statistical Modeling

**DOI:** 10.3390/diagnostics16121779

**Published:** 2026-06-09

**Authors:** Yeliz Çeçen Dönmez, Esra Keles, İsmail Bağlar, Fatih Şanlıkan, Sahra Sultan Kara, Öznur Dündar Akin, Naile Fevziye Misirlioglu, Seyma Dumur, Hafize Uzun

**Affiliations:** 1 Department of Obstetrics and Gynecology, University of Health Sciences, Kartal Dr. Lütfi Kırdar City Hospital, Istanbul 34865, Turkey; yelizcecendonmez@gmail.com (Y.Ç.D.); ismailbg@gmail.com (İ.B.); sahracavusoglu@gmail.com (S.S.K.); 2 Department of Gynecologic Oncology, University of Health Sciences, Kartal Dr. Lütfi Kırdar City Hospital, Istanbul 34865, Turkey; dresrakeles@hotmail.com (E.K.); fatihsanlikan1@gmail.com (F.Ş.); 3 Department of Obstetrics and Gynecology, Faculty of Medicine, Istanbul Atlas University, Istanbul 34408, Turkey; dr.oznurdundar83@gmail.com; 4 Department of Biochemistry, Faculty of Medicine, Istanbul Atlas University, Istanbul 34408, Turkey; nailemisirlioglu@gmail.com (N.F.M.); seyma.dumur@atlas.edu.tr (S.D.)

**Keywords:** gestational diabetes mellitus, Wnt signaling, WIF-1, secreted frizzled-related protein, beta-catenin-1

## Abstract

**Objectives:** Gestational diabetes mellitus (GDM) is a common metabolic disorder characterized by insulin resistance and systemic inflammation. Emerging evidence suggests that the Wnt/β-catenin signaling pathway may play a role in metabolic dysregulation; however, its clinical relevance in GDM remains unclear. This study aimed to evaluate the diagnostic value of Wnt signaling-related biomarkers, including Wnt-inhibitory factor 1 (WIF-1), secreted frizzled-related protein-4 (SFRP-4), and beta-catenin-1 (CTNNB1) in GDM. **Methods:** This case–control study included 60 patients with GDM and 60 healthy pregnant controls. Serum levels of WIF-1, SFRP-4, and CTNNB1 were measured and compared between groups. Receiver operating characteristic (ROC) and multivariable logistic regression assessed diagnostic performance and predictors, while correlation analysis and principal component analysis (PCA) evaluated biomarker relationships. **Results:** Serum levels of WIF-1, SFRP4, and CTNNB1 were significantly higher in the GDM group (all *p* < 0.001). ROC analysis showed moderate diagnostic performance for individual biomarkers, with CTNNB1 demonstrating the highest discriminative ability. The combined biomarker model significantly improved diagnostic accuracy, yielding the highest area under the curve (AUC), sensitivity, and specificity. In multivariable analysis, all three biomarkers remained independently associated with GDM. Correlation analysis revealed moderate interrelationships, with SFRP4 acting as a central component. PCA demonstrated partial separation between GDM and control groups, supporting the ability of Wnt signaling-related biomarkers to capture disease-associated biological variation. **Conclusions:** Wnt signaling-related biomarkers, including WIF-1, SFRP4, and CTNNB1, are significantly elevated in GDM and show promising diagnostic value. The combined biomarker approach provides superior discriminative performance compared to individual markers, highlighting its potential role in improving risk stratification and personalized management.

## 1. Introduction

Gestational diabetes mellitus (GDM) is a prevalent metabolic complication of pregnancy characterized by glucose intolerance with onset during gestation. It is associated with increased risks of maternal and fetal complications, as well as long-term disorders such as type 2 diabetes (T2DM) and cardiovascular disease [1]. Current management strategies include lifestyle modification, glucose monitoring, and pharmacological treatment when glycemic targets cannot be achieved through dietary interventions alone [2]. Despite advances in screening, early diagnosis remains suboptimal, necessitating the discovery of novel biomarkers and mechanistic insights [1].

Recent research has focused on the Wnt/β-catenin signaling pathway as a primary regulator of metabolic homeostasis. This pathway is critical for glucose metabolism, lipid regulation, pancreatic β-cell proliferation, insulin secretion, and insulin sensitivity. Mechanistically, activation of Wnt signaling stabilizes beta-catenin-1 (CTNNB1), which subsequently regulates the transcription of genes involved in glucose homeostasis and metabolic control. Emerging evidence suggests that dysregulation of the Wnt/β-catenin pathway contributes to insulin resistance, chronic inflammation, oxidative stress, and β-cell dysfunction, which are central pathophysiological features of GDM. In addition, aberrant activation of Wnt signaling has been implicated in placental trophoblast dysfunction and impaired glucose transport under hyperglycemic conditions during pregnancy [3,4,5,6]. Dysregulation of this pathway is implicated in obesity and GDM and may contribute to impaired glucose regulation during pregnancy [3,4].

The activity of this pathway is modulated by key extracellular antagonists. Wnt-inhibitory factor 1 (WIF-1) binds directly to Wnt ligands and suppresses downstream β-catenin signaling activity [7]. Notably, WIF-1 has been shown to ameliorate mitochondrial dysfunction in diabetic retinopathy through AMPK/mTOR pathway-mediated mechanisms [8]. Secreted frizzled-related protein-4 (sFRP4), identified as a key gene in T2DM-related networks within human pancreatic islets, modulates Wnt signaling through competitive binding. Increased SFRP4 expression is associated with β-cell dysfunction and elevated circulating levels in T2DM patients, although its exact relationship with diabetic complications remains unclear. Although evidence remains limited, several recent studies have suggested that elevated circulating SFRP4 levels may predict impaired glucose tolerance and GDM development, supporting its potential role as an early biomarker of metabolic dysregulation during pregnancy [9,10,11,12].

**Figure 1:**
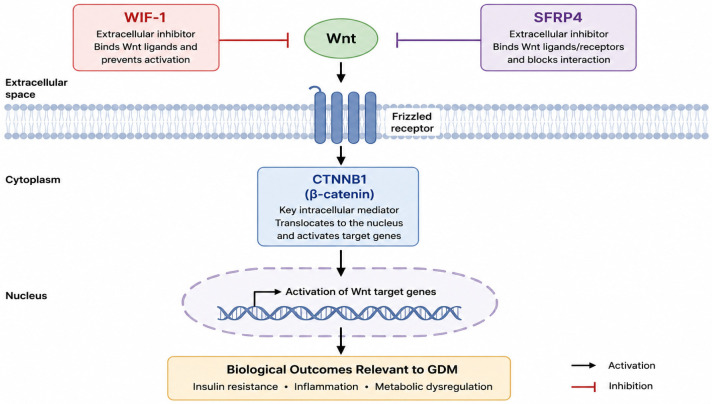
Roles of WIF-1, SFRP4, and CTNNB1 in the Wnt/β-catenin pathway and GDM.

Despite these findings, the clinical relevance of circulating Wnt pathway-related biomarkers in GDM remains insufficiently explored. In particular, the roles of WIF-1, SFRP4, and CTNNB1 as diagnostic biomarkers have not been comprehensively evaluated in a clinical setting. Moreover, these biomarkers were specifically selected because they represent distinct but complementary components of the Wnt/β-catenin signaling cascade. WIF-1 functions as an extracellular Wnt antagonist by binding Wnt ligands and suppressing downstream β-catenin signaling activity, whereas SFRP4 modulates ligand–receptor interactions and has been associated with β-cell dysfunction, insulin resistance, and impaired glucose metabolism. In contrast, CTNNB1 serves as the central intracellular effector of canonical Wnt signaling and plays a critical role in glucose homeostasis, insulin signaling, and metabolic regulation. Previous experimental and clinical studies have demonstrated associations between these molecules and diabetes-related metabolic dysfunction, supporting their potential relevance in the pathophysiology of GDM [7,8,9,10,11,12,13]. To our knowledge, previous clinical studies have primarily focused on individual Wnt- related biomarkers, particularly SFRP4, in GDM and T2DM [7,8,9,10,11,12,13], whereas the simultaneous evaluation of WIF-1, SFRP4, and CTNNB1 in patients with GDM remains limited. A schematic overview of the interactions among WIF-1, SFRP4, and CTNNB1 within the Wnt/β-catenin signaling pathway is presented in Figure 1.

The present study aimed to investigate the serum levels of Wnt signaling-related biomarkers (WIF-1, sFRP4, and CTNNB1) in patients with GDM and to evaluate their diagnostic performance using integrative statistical modeling approaches.

## 2. Materials and Methods

### 2.1. Ethical Approval

The study protocol was approved by the Kartal Koşuyolu High Specialization Training and Research Hospital under approval number 2025/21/1276; dated 18 November 2025. Written informed consent was obtained from all participants before enrollment. The study was conducted in accordance with the ethical principles of the Declaration of Helsinki.

### 2.2. Study Design and Participants

This case–control study was conducted in pregnant women attending the obstetrics outpatient clinic of Kartal Dr. Lütfi Kırdar City Hospital. The study population consisted of women diagnosed with GDM and healthy pregnant controls with comparable age distribution. In the present cohort, 60 women with GDM and 60 controls were included.

Eligible participants were singleton pregnancies evaluated during the second trimester. Women with pregestational T1DM or T2DM, multifetal gestation, active infection, chronic inflammatory disease, autoimmune disease, malignancy, severe renal or hepatic dysfunction, or use of medications known to affect glucose metabolism were excluded. Controls were selected from pregnant women with normal glucose tolerance and no major obstetric or systemic complications.

### 2.3. Diagnosis of GDM

GDM was diagnosed using a 75 g oral glucose tolerance test (OGTT) performed at 24–28 weeks of gestation. Diagnostic thresholds followed the International Association of Diabetes and Pregnancy Study Groups (IADPSG)/WHO 2013 framework: fasting plasma glucose ≥ 5.1 mmol/L, 1 h plasma glucose ≥ 10.0 mmol/L, or 2 h plasma glucose ≥ 8.5 mmol/L, with one or more abnormal values considered diagnostic. These thresholds are the basis of widely used modern GDM classification schemes and remain the most common peer-reviewed reference standard in contemporary observational work [10,11].

All patients diagnosed with GDM received standard obstetric and endocrinological care according to institutional clinical practice. Management included dietary counseling, lifestyle modification, and self-monitoring of blood glucose levels. Pharmacological treatment, including insulin therapy when indicated, was initiated into patients who failed to achieve glycemic targets through lifestyle interventions alone. Clinical management was consistent with contemporary recommendations for the prevention and treatment of GDM as described by Chatzakis et al. [2].

### 2.4. Clinical and Biochemical Data Collection

Maternal age, gravidity, parity, gestational age at sampling, anthropometric characteristics, smoking status, and relevant medical history were recorded from the clinical database and medical files at the time of enrollment. Routine laboratory parameters, including fasting glucose, OGTT values, HbA1c, insulin, C-reactive protein (CRP), complete blood count, and standard biochemical markers, were obtained from fasting venous blood samples collected on the same day as clinical evaluation. Hemoglobin levels, white blood cell counts, platelet counts, and related clinical parameters were evaluated to better characterize the hematological and inflammatory profile of the study population and to minimize potential confounding effects. All laboratory measurements, including WIF-1, SFRP4, and CTNNB1 levels, were obtained from fasting venous blood samples collected during the second trimester at the time of routine GDM screening (24–28 weeks of gestation).

### 2.5. Blood Sampling, Serum Processing and Laboratory Measurements

After an overnight fast of at least 8 h, venous blood samples were drawn from all participants between 08:00 and 10:00 a.m. Samples intended for biomarker analysis were allowed to clot, centrifuged at 3000 rpm for 10 min, and the separated serum aliquoted and stored at −80 °C until analysis. Repeated freeze–thaw cycles were avoided. This preanalytical workflow is in line with standard handling procedures reported in clinical biomarker studies of GDM and related pregnancy cohorts.

### 2.6. Measurement of Serum Wnt Signaling-Related Biomarkers

Serum concentrations of WIF-1, SFRP4, and CTNNB1 were measured using commercially available sandwich enzyme-linked immunosorbent assay (ELISA) kits (BT Lab, Shanghai, China) according to the manufacturer’s instructions. The WIF-1 assay (Cat. No: E2361Hu) had a detection range of 20–6000 ng/L and a sensitivity of 10.12 ng/L, with intra-assay coefficients of variation (CVs) ranging from 2.39% to 4.98% and inter-assay CVs < 10%. The SFRP4 assay (Cat. No: E2327Hu) had a detection range of 0.05–15 ng/mL and a sensitivity of 0.024 ng/mL, with intra-assay CVs ranging between 5.4% and 6.8% and inter-assay CVs < 10%. The CTNNB1 assay (Cat. No: E2396Hu) had a detection range of 10–2000 ng/L and a sensitivity of 5.08 ng/L, with intra-assay CVs ranging from 2.4% to 4.9% and inter-assay CVs < 10%. All serum samples were analyzed in duplicate, and biomarker concentrations were calculated using standard calibration curves.

### 2.7. Statistical Analysis

All analyses were performed using SPSS 19.0 statistical software (SPSS, Chicago, IL, USA). Continuous variables were assessed for normality using the Shapiro–Wilk test and visual inspection of histograms and Q–Q plots. Normally distributed variables are presented as mean ± standard deviation, whereas non-normally distributed variables are presented as median [minimum–maximum]. Categorical variables are reported as number (percentage). Between-group comparisons were performed using the independent-samples Student’s *t*-test or Mann–Whitney U test for continuous variables, and the chi-square test or Fisher’s exact test for categorical variables, as appropriate. Receiver operating characteristic (ROC) curve analysis was used to evaluate the diagnostic performance of WIF-1, sFRP4, and CTNNB1 for identifying GDM. The area under the curve (AUC) with 95% confidence intervals was calculated for each biomarker. Optimal cut-off values were determined using the Youden index. When ROC curves were compared, DeLong’s nonparametric method was used. These are standard, peer-reviewed approaches for evaluating diagnostic biomarkers. To identify predictors of GDM, univariable logistic regression analyses were first performed for clinically relevant variables and biomarker levels. Variables with clinical relevance and/or statistical significance in univariable analysis were entered into a multivariable logistic regression model. Odds ratios (ORs) and 95% confidence intervals (CIs) were reported. If missing data were handled using multiple imputations, estimates should be pooled according to Rubin’s rules; multiple imputation by chained equations (MICE) is a well-established framework for this purpose. A post hoc power analysis indicated that the present sample size provided adequate statistical power (>80%) to detect significant differences between the study groups at a two-sided significance level of 0.05. A two-sided *p* value < 0.05 was considered statistically significant.

## 3. Results

The demographic and clinical characteristics of the study population are summarized in Table 1. Maternal age and gestational age at sampling were comparable between the groups (*p* > 0.05 for both), confirming similar age distribution between the GDM and control groups. However, gravida and parity were significantly higher in the GDM group compared to controls (*p* = 0.031 and *p* = 0.024, respectively). In addition, body weight and body mass index (BMI) were significantly increased in patients with GDM (*p* = 0.006 and *p* < 0.001, respectively). No statistically significant differences were observed in abortion history, height, or smoking status (*p* > 0.05 for all). Previous GDM was significantly more frequent in the GDM group (*p* = 0.004).

Biochemical, metabolic, and hematological parameters of the study population are summarized in Table 2. Fasting glucose and OGTT values (both 1 h and 2 h) were significantly higher in the GDM group (all *p* < 0.001). Similarly, HbA1c and insulin levels were significantly elevated in patients with GDM (*p* < 0.001 and *p* = 0.006, respectively). CRP levels were also markedly increased in the GDM group, indicating enhanced systemic inflammation (*p* = 0.0005). In contrast, HOMA-IR values showed no statistically significant difference between groups (*p* = 0.081). Liver function tests, total protein, albumin, and most hematological parameters were comparable between groups (*p* > 0.05). A small but statistically significant increase in creatinine levels was observed in the GDM group (*p* = 0.035).

Serum levels of Wnt signaling-related biomarkers are presented in Table 3. WIF1, SRF4 and CTNNB1 levels were significantly higher in the GDM group compared to controls (all *p* < 0.001). Specifically, mean WIF-1 levels were markedly elevated in patients with GDM, accompanied by significant increases in SFRP4 and CTNNB1 concentrations. These findings suggest activation of Wnt/β-catenin-related signaling pathways in GDM.

**Figure 2:**
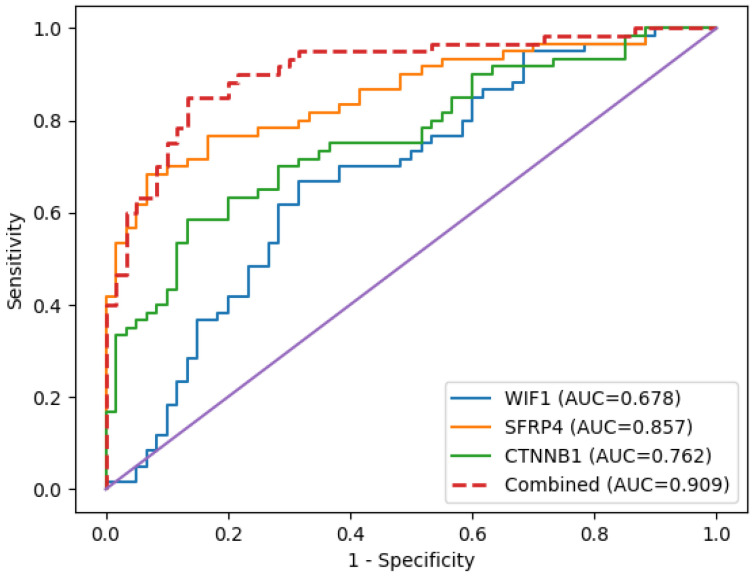
Diagnostic performance of serum Wnt-inhibitory factor 1 (WIF1), secreted frizzled-related protein-4 (SFRP4), beta-catenin-1 (CTNNB1) for identifying gestational diabetes mellitus.

The diagnostic performance of WIF1, SRF4 and CTNNB1 is summarized in Table 4, and the corresponding ROC curves are presented in Figure 1. All three biomarkers demonstrated moderate diagnostic performance when evaluated individually. Among them, CTNNB1 exhibited the highest discriminative ability, followed by WIF1 and SRF4. Importantly, the combined biomarker model showed superior diagnostic performance compared to individual markers, as illustrated in Figure 2. The combined model achieved the highest AUC, sensitivity, and specificity, indicating that integration of these biomarkers significantly improves diagnostic accuracy.

The results of logistic regression analyses are presented in Table 5. Univariable analysis identified insulin, CRP, WIF1, SRF4 and CTNNB1 as significant predictors of GDM (all *p* < 0.01). In the multivariable model, WIF1 (*p* = 0.019), SRF4 (*p* = 0.004), and CTNNB1 (*p* = 0.004) remained independently associated with GDM. Insulin remained independently associated with GDM after multivariable adjustment (*p* = 0.008), whereas age, BMI, and HbA1c did not retain statistical significance (*p* > 0.05).

A moderate positive correlation was observed between WIF1 and SFRP4 (r ≈ 0.33, *p* < 0.05). Similarly, weak SFRP4 was positively correlated with CTNNB1 (r ≈ 0.26, *p* < 0.05). In contrast, no significant correlation was found between WIF1 and CTNNB1 (*p* > 0.05). Overall, these findings suggest partial interdependence among Wnt pathway components, with SFRP4 appearing as a central interacting node linking upstream inhibition (WIF1) and downstream signaling (CTNNB1). Correlation analysis demonstrated moderate interrelationships among WIF-1, SFRP4, and CTNNB1 levels, supporting the possibility of coordinated dysregulation within the Wnt/β-catenin signaling pathway in GDM. These results support the hypothesis that Wnt signaling dysregulation in GDM may involve coordinated but not fully overlapping biomarker interactions.

**Figure 3:**
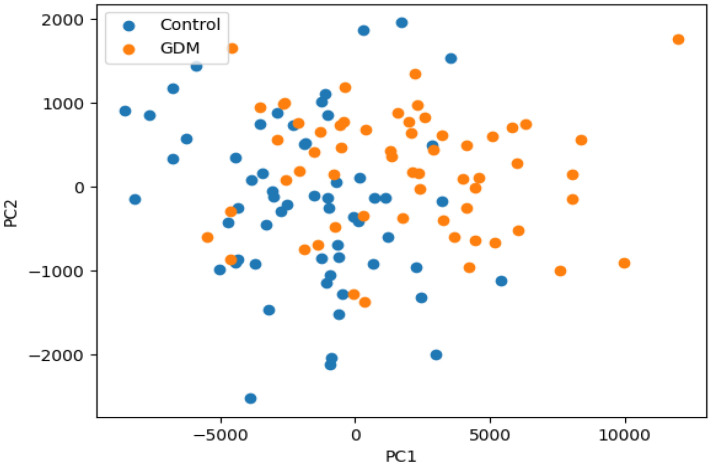
Principal component analysis (PCA) of Wnt Signaling-Related Biomarkers in Gestational Diabetes Mellitus.

Principal component analysis (PCA) illustrates the distribution and clustering of control and GDM groups based on Wnt signaling-related biomarkers (WIF1, SFRP4, and CTNNB1) (Figure 3). The observed separation pattern indicates that these biomarkers capture underlying biological variability associated with GDM, although partial overlap suggests shared metabolic characteristics between groups.

## 4. Discussion

In the present study, serum WIF1, SFRP4, and CTNNB1 levels were significantly elevated in women with GDM compared to controls, and the combined biomarker model demonstrated superior diagnostic performance over individual markers. Among these markers, the observed increase in SFRP4 is consistent with previous studies reporting elevated circulating SFRP4 levels in GDM and its association with impaired glucose metabolism and β-cell dysfunction [12,13]. In contrast, although Wnt/β-catenin signaling has been widely implicated in metabolic regulation and diabetes-related pathways, clinical studies specifically assessing circulating WIF1 and CTNNB1 in GDM remain extremely limited or absent, suggesting that these biomarkers may represent underexplored components of disease pathophysiology.

GDM is one of the most common metabolic disorders in pregnancy and is characterized by increased production of proinflammatory and antiangiogenic factors that impair trophoblast function, including proliferation, migration, and invasion, thereby adversely affecting maternal and fetal outcomes [14]. Additionally, offspring of mothers with GDM are at increased risk of developing T2DM and metabolic syndrome later in life [15]. Although the underlying mechanisms remain incompletely understood, impaired placental glucose transport has been implicated in this process [16].

The observed elevation of Wnt signaling-related biomarkers in GDM may be explained by the central role of the Wnt/β-catenin pathway in metabolic regulation and pancreatic β-cell function. Under physiological conditions, Wnt signaling contributes to insulin secretion, β-cell proliferation, and glucose homeostasis [17,18,19,20]. However, dysregulation of this pathway has been associated with impaired insulin signaling, increased inflammation, and β-cell dysfunction, which are key features of GDM [6]. In this context, β-catenin interacts with molecules such as MUC1, which has been shown to activate Wnt/β-catenin signaling and contribute to trophoblast dysfunction during pregnancy in GDM [4,12,13]. Although the absolute difference in HbA1c levels between the groups was modest, the observed increase in the GDM group was statistically significant and may reflect clinically relevant alterations in glycemic regulation during pregnancy.

Interestingly, all three biomarkers, including the Wnt antagonists WIF-1 and SFRP4 as well as the downstream signaling effector CTNNB1, were elevated in patients with GDM. Although this finding may initially appear paradoxical, it may reflect compensatory feedback activation or incomplete inhibitory regulation within the Wnt/β-catenin signaling pathway under chronic metabolic stress conditions. In addition, circulating concentrations of WIF-1, SFRP4, and CTNNB1 may not directly correspond to intracellular signaling activity, as Wnt pathway regulation is highly dynamic and context dependent. Therefore, the simultaneous elevation of inhibitory and downstream signaling components may represent dysregulated pathway homeostasis rather than simple pathway suppression. Furthermore, several downstream mediators of the Wnt/β-catenin pathway, including TCF7L2, GSK3β, and c-Myc, have also been implicated in insulin resistance, β-cell dysfunction, and glucose metabolism [3,4,6], supporting the complex and multifactorial nature of Wnt signaling dysregulation in GDM.

SFRP4 has been shown to interfere with Wnt signaling and impair insulin secretion, thereby contributing to β-cell dysfunction and glucose intolerance. Consistent with this, elevated circulating SFRP4 levels have been reported in individuals with GDM and linked to metabolic dysregulation, including early pregnancy prediction models [8,9,12,13]. Although evidence on maternal circulating SFRP4 in GDM remains limited, it represents one of the few Wnt-related biomarkers supported by clinical data. Previous studies have also suggested functional interactions among extracellular Wnt antagonists and downstream β-catenin signaling components in metabolic disorders [6,13]. In the present study, moderate correlations among WIF1, SFRP4, and CTNNB1 levels support the possibility of coordinated regulation within the Wnt/β-catenin signaling pathway in GDM. However, the absence of strong correlations between all biomarkers may indicate partially independent regulatory mechanisms and the multifactorial nature of Wnt pathway dysregulation during pregnancy.

Furthermore, aberrant activation of the Wnt/β-catenin pathway has been demonstrated in trophoblast models and placental tissues under hyperglycemic conditions, suggesting a role in placental dysfunction in GDM. Although direct clinical evidence regarding circulating WIF1 and CTNNB1 levels remains limited, both molecules are integral components of the Wnt signaling cascade, with WIF1 acting as an extracellular antagonist and CTNNB1 serving as the central intracellular effector [6,12,13,21]. Recent studies have also highlighted the involvement of SFRP4 in obesity and T2DM [22,23,24]. In addition, experimental evidence indicates that SFRP4 expression is dynamically regulated during pregnancy, with peak expression in early to mid-gestation followed by a decline in later stages, suggesting a temporally controlled role in placental function [25,26].

Importantly, our findings demonstrate that a multimarker model provides superior diagnostic performance compared to individual biomarkers, reflecting the multifactorial nature of GDM. While single markers capture isolated aspects of pathophysiology, integrating WIF1, SFRP4, and CTNNB1 enables a more comprehensive assessment of the Wnt signaling cascade, spanning extracellular inhibition, receptor modulation, and intracellular signal transduction. Correlation and network analyses suggest coordinated dysregulation of these components, with SFRP4 potentially acting as a central mediator. From a clinical perspective, this aligns with evidence that integrative approaches outperform single biomarkers in complex metabolic diseases, improving early detection and risk stratification. Metabolic factors such as BMI, obesity, insulin resistance, gestational age, and lipid abnormalities may also influence circulating Wnt signaling-related biomarker levels. Previous studies have demonstrated associations between SFRP4 and obesity, insulin resistance, and impaired glucose metabolism, suggesting that these metabolic parameters may contribute to Wnt pathway dysregulation in GDM [8,9,10]. A summary of previous studies investigating Wnt-related biomarkers in GDM and T2DM is presented in Table 6.

Principal component analysis (Figure 3) demonstrated partial separation between GDM and control groups, indicating that Wnt signaling-related biomarkers capture relevant biological variation. However, the observed overlap suggests that individual biomarkers may be insufficient alone, further supporting the superior performance of the combined biomarker model.

### 4.1. Strengths

This study has several notable strengths. It is among the first to comprehensively evaluate Wnt signaling-related biomarkers (WIF1, SFRP4, and CTNNB1) simultaneously in GDM. The integration of ROC analysis and multivariable modeling enhances the robustness and clinical relevance of the findings. In addition, the multimarker approach provides a comprehensive assessment of the Wnt signaling cascade, while correlation analyses offer mechanistic insight into biomarker interactions.

### 4.2. Limitations

Several limitations should be acknowledged. The relatively modest sample size and single-center design may limit generalizability. In addition, the absence of an independent validation cohort may limit the external reproducibility of the findings. The cross-sectional design precludes causal inference and does not allow assessment of temporal changes during pregnancy. Although SFRP4 has been previously studied, clinical data regarding WIF1 and CTNNB1 remain limited, requiring external validation. Additionally, potential confounders such as lifestyle and genetic factors were not fully evaluated, and mechanistic validation was not performed. Moreover, multiple ROC, correlation, and regression analyses were performed without formal adjustment for multiple comparisons, which may increase the risk of type I error. Furthermore, the present study evaluated circulating protein levels of WIF1, SFRP4, and CTNNB1 using ELISA-based measurements, whereas gene expression analyses were not performed. Therefore, potential associations between transcriptional regulation and circulating protein levels could not be assessed. All biomarker measurements were performed using ELISA kits from a single manufacturer, which may introduce potential assay-related or batch-related variability despite standardized analytical procedures. Future studies integrating both gene expression and proteomic analyses may provide a more comprehensive understanding of Wnt/β-catenin pathway dysregulation in GDM. Given the cross-sectional design of the study, the findings should be interpreted as diagnostic associations rather than predictive relationships.

### 4.3. Clinical Implications

Wnt signaling-related biomarkers may serve as promising tools for improving the diagnosis and risk stratification of GDM. The superior performance of the combined biomarker model suggests that a multimarker approach better reflects the complex pathophysiology of GDM. Integration into clinical practice may enhance early detection and support personalized management strategies, while also highlighting potential therapeutic targets within the Wnt/β-catenin pathway. Elevated WIF1, SFRP4, and CTNNB1 levels were associated with adverse metabolic features observed in GDM, including impaired glucose regulation, insulin resistance, and altered inflammatory status. These alterations may reflect dysregulation of the Wnt4/β-catenin signaling pathway, which plays a role in placental function, β-cell homeostasis, and glucose metabolism during pregnancy.

## 5. Conclusions

Wnt signaling-related biomarkers, including WIF1, SFRP4, and CTNNB1, are significantly elevated in GDM and demonstrate meaningful diagnostic potential. The combined biomarker model provides superior discriminative performance compared to individual markers, supporting the value of integrative approaches in complex metabolic disorders. Further large-scale and mechanistic studies are warranted to validate these findings.

## Figures and Tables

**Table 1:** Baseline maternal demographic and clinical characteristics of the study groups.

Variable	Control (*n* = 60)	GDM (*n* = 60)	*p* Value
**Age (years)**	29.4 ± 5.8	31.6 ± 5.1	0.065
**Gestational age at sampling (weeks)**	26.5 [24–38]	28.2 [16–34]	0.088
**Gravida** ***n*** **(range)**	2 [1–7]	3 [1–9]	0.031
**Parity** ***n*** **(range)**	1 [0–3]	2 [0–5]	0.024
**Height (cm)**	160 [150–172]	158 [154–171]	0.398
**Weight (kg)**	71.8 ± 10.2	79.3 ± 13.8	0.006
**Body mass index (kg/m^2^)**	27.22 ± 3.61	30.71 ± 5.18	<0.001
**Smoking,** ***n*** **(%)**	9 (15.0)	5 (8.3)	0.214
Previous GDM, ***n*** **(%)**	0 (0)	8 (13.3)	0.004

**Continuous variables:** Student’s *t*-test or Mann–Whitney U test; categorical: χ^2^ test.

**Table 2:** Biochemical, metabolic, and hematological parameters of the study groups.

Variable	Control (*n* = 60)	GDM (*n* = 60)	*p* Value
**Fasting glucose (mg/dL)**	85 [72–97]	101 [78–221]	<0.001
**OGTT, 1 h (mg/dL)**	134.2 ± 22.8	168.9 ± 35.4	<0.001
**OGTT, 2 h (mg/dL)**	103.5 ± 21.3	139.1 ± 32.6	<0.001
**HbA1c (%)**	5.4 [4.5–5.9]	5.9 [5.4–7.8]	<0.001
**Insulin (µIU/mL)**	6.2 [4.4–77.1]	14.8 [6.9–129.7]	0.006
**HOMA-IR**	1.2 [0.3–7.6]	2.4 [0.4–32.3]	0.081
**C-reactive protein (mg/L)**	2.1 [0.1–4.2]	12.4 [0.2–43.6]	<0.001
**Total protein (g/dL)**	6.5 [5.8–7.4]	6.4 [5.7–7.3]	0.842
**Albumin (g/dL)**	3.7 [3.1–4.6]	3.7 [3.0–4.6]	0.571
**AST (U/L)**	17.1 [11–37]	19.2 [13–39]	0.441
**ALT (U/L)**	13.8 [9–32]	14.3 [10–41]	0.901
**Hemoglobin (g/dL)**	11.4 ± 1.3	11.6 ± 1.3	0.372
**White blood cell count (/µL)**	9.3 ± 2.4	9.8 ± 2.7	0.341
**Platelet count (/µL)**	252 ± 60	245 ± 75	0.128

Continuous values are presented as mean ± standard deviation or median [minimum–maximum].

**Table 3:** Serum WIF1, SRF4 and CTNNB1 levels in the study groups.

Variable	Control (*n* = 60)	GDM (*n* = 60)	*p* Value
**WIF1 (ng/mL)**	2.65 ± 8.80	3.36 ± 7.30	<0.001
**SFRP4 (ng/mL)**	6.70 ± 2.40	9.50 ± 2.80	<0.001
**CTNNB1 (ng/mL)**	8.70 ± 2.70	12.75 ± 3.20	<0.001

**WIF1,** Wnt-inhibitory factor 1; **SFRP4**, Secreted frizzled-related protein-4; **CTNNB1,** beta-catenin-1.

**Table 4:** Diagnostic performance of serum biomarkers.

Biomarker	AUC (95% CI)	Cut-Off	Sensitivity (%)	Specificity (%)	PPV (%)	NPV (%)	*p* Value
**WIF-1**	0.735 (0.64–0.82)	3.09	67.0	71.0	70.5	67.4	<0.001
**SFRP4**	0.738 (0.63–0.83)	8.10	71.0	69.0	70.8	69.3	<0.001
**CTNNB1**	0.805 (0.71–0.89)	10.10	83.0	76.0	78.2	81.1	<0.001
**Combined model**	0.910 (0.84–0.96)	0.42	92.0	84.0	86.5	90.5	<0.001

AUC comparisons performed using DeLong test. **AUC:** Area under the curve; **CI:** Confidence interval; **PPV:** Positive predictive value; **NPV:** Negative predictive value; **WIF1,** Wnt-inhibitory factor 1; **SFRP4**, Secreted frizzled-related protein-4; **CTNNB1,** beta-catenin-1.

**Table 5:** Logistic regression analysis for predictors of gestational diabetes mellitus.

Variable	Univariable OR (95% CI)	*p* Value	Multivariable OR (95% CI)	*p* Value
**Age (Year)**	0.909 (0.635–1.301)	0.603	2.322 (0.714–7.548)	0.161
**BMI (kg/m^2^)**	0.888 (0.619–1.273)	0.518	0.574 (0.269–1.228)	0.153
**HbA1c (%)**	1.273 (0.879–1.844)	0.202	1.201 (0.474–3.047)	0.700
**Insulin (µIU/mL)**	3.415 (1.821–6.406)	<0.001	23.218 (2.284–236.067)	0.008
**CRP (mg/L)**	0.055 (0.019–0.157)	<0.001	0.003 (0.000–0.076)	<0.001
**WIF1 (ng/L)**	1.939 (1.283–2.930)	0.002	4.877 (1.291–18.426)	0.019
**SFRP4 (ng/mL)**	6.780 (3.336–13.776)	<0.001	5.479 (1.747–17.186)	0.004
**CTNNB1 (ng/L)**	3.367 (1.997–5.678)	<0.001	8.794 (2.002–38.625)	0.004

**OR,** odds ratio; **CI,** confidence interval. Odds ratios are expressed per 1 standard deviation increase in each variable. The multivariable model included age, BMI, HbA1c, insulin, CRP, WIF1, SFRP4, and CTNNB1 simultaneously. **BMI,** Body mass index; **CRP,** C-reactive protein; **WIF1,** Wnt-inhibitory factor 1; **SFRP4**, Secreted frizzled-related protein-4; **CTNNB1,** beta-catenin-1.

**Table 6:** Summary of previous studies investigating wnt-related biomarkers in GDM and T2DM.

Author/Year	Disease	Biomarker(s)	Sample Type	Main Findings
**Jin, 2008** [3]	T2DM	Wnt/β-catenin pathway	Review article	Dysregulation of Wnt signaling contributes to impaired glucose metabolism, insulin resistance, and β-cell dysfunction.
**Sánchez-Pozos et al., 2024** [8]	Prediabetes/T2DM	SFRP4	Serum	Elevated circulating SFRP4 levels were associated with impaired glucose metabolism and insulin resistance.
**Schuitemaker et al., 2020** [12]	GDM	SFRP4	Maternal serum	Increased first-trimester SFRP4 concentrations were associated with subsequent development of GDM.
**Yuan et al., 2018** [13]	GDM	SFRP4, Ficolin-3	Serum	Women with GDM exhibited significantly elevated circulating SFRP4 levels compared to controls.
**Cui et al., 2023** [4]	GDM	Wnt/β-catenin pathway	Placental trophoblast model	Dysregulated Wnt/β-catenin signaling contributed to trophoblast dysfunction under hyperglycemic conditions.
**Zou et al., 2022** [7]	Diabetic complications	WIF1	Experimental model	WIF1 modulated AMPK/mTOR signaling and improved mitochondrial dysfunction associated with diabetes.
**Present study**	GDM	WIF1, SFRP4, CTNNB1	Serum	Simultaneous evaluation of circulating Wnt-related biomarkers demonstrated altered pathway activity and improved diagnostic performance using an integrated multimarker approach.

## Data Availability

The datasets used and/or analyzed during the current study are available from the corresponding author on reasonable request.
